# Methylmercury promotes breast cancer cell proliferation

**DOI:** 10.1016/j.toxrep.2018.05.002

**Published:** 2018-05-25

**Authors:** Hilary M. Gaudet, Emily Christensen, Brandon Conn, Sara Morrow, Lauren Cressey, Janina Benoit

**Affiliations:** Department of Chemistry, Wheaton College, Norton, MA, USA

**Keywords:** Methylmercury, Mercury, Metalloestrogen, Breast cancer

## Abstract

**Context:**

Metalloestrogens are small ionic metals that activate the estrogen receptor (ER). Studies have shown that when metalloestrogens bind to the ER, there is an increase in transcription and expression of estrogen-regulated genes, which induces proliferation of estrogen-dependent breast cancer. Methylmercury (MeHg), a metalloestrogen, is present in the environment and is toxic at moderate to high concentrations. However, at lower concentrations MeHg may promote the proliferation of ER-positive breast cancers and protect cells against pro-apoptotic signals.

**Objective:**

To investigate the effects of MeHg treatment on breast cancer cells in vitro.

**Materials and methods:**

MCF7 breast cancer cells were treated with concentrations of MeHg ranging from 1 nM to 100 mM. Hg analysis was used to quantify intracellular mercury concentrations. Cell proliferation and apoptosis were determined by cell counting and Annexin-V staining, respectively.

**Results:**

We defined a protocol that maximizes cellular exposure to mercury. Treatment of human ER-positive breast cancer cells with 1 nM MeHg promoted proliferation, while treatment with a concentration of 100 nM induced apoptosis.

**Discussion and conclusions:**

Clarifying the effects of MeHg on breast cancer will improve our understanding of how environmental toxins affect tumor progression and may lead to the development of future therapeutic strategies.

## Highlights

• More mercury is retained in cell supernatant when exposed in full versus minimal media.
• More mercury associates with cells exposed in minimal media compared to full media.
• Less mercury promotes breast cancer cell proliferation when exposed in minimal media.

## Graphical abstract

**Figure:**
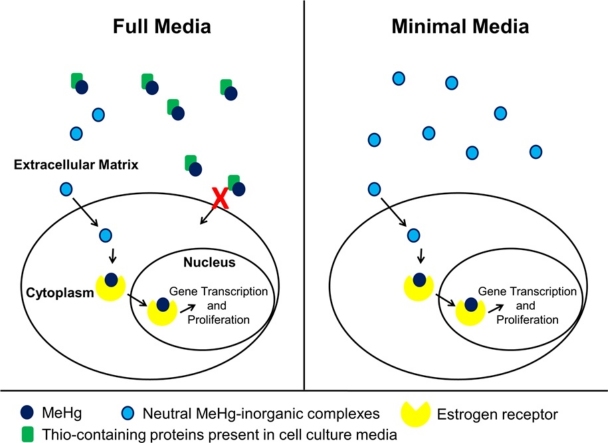

## 1 Introduction

Breast cancer is the second most common cancer diagnosis in women in the United States [1]. It accounts for one in three cancer diagnoses and is the second leading cause of cancer death [1]. Estrogens are a family of steroid hormones that directly control the expression of cell-cycle regulatory genes [2]. Breast cancer is associated with elevated levels of estrogen or estrogen-like substances that bind to the estrogen receptor (ER), causing overstimulation of signaling pathways [3].

The high incidence of breast cancer is likely, in part, due to the presence of environmental estrogens [4,5]. Environmental estrogens such as phytoestrogens or plant-based estrogens (coumestrol and isoflavone genistein) and xenoestrogens or synthetic chemicals (dichlorodiphenyltrichloroethane, bisphenol A, phthalates, dichlorodiphenylethylene, polychlorinated biphenyls, and alkylphenol) have been shown to promote estrogen-like effects [[6], [7], [8]]. These environmental estrogens can be found in plants, pesticides, birth control pills, plastics, auto exhaust, and cigarette smoke [4,[9], [10], [11]]. Recently, several inorganic xenoestrogens—metalloestrogens—have been shown to mimic the effect of estrogens and activate the ER [4,5,11]. Metalloestrogens are small ionic metals and metalloids that fall into two subcategories, oxyanions and bivalent cations [4,12,13]. The oxyanions include arsenite, antimony, nitrite, selenite, and vanadate, while the bivalent cations include cadmium, calcium, colbolt, copper, nickel, chromium, lead, mercury, and tin [4].

Copper, colbolt, nickel, lead, tin, and chromium (II) have been shown to induce the proliferation of ER-positive breast cancer cells [4,5,11,14] and increase the transcription and expression of estrogen-regulated genes [4,5]. These metals have also been shown to bind with high affinity to the ER and block the binding of estradiol [13]. Among the heavy metals, cadmium and mercury are two of the most toxic due to their persistence in the environment [15]. Both have been shown to cause oxidative stress and induce apoptosis [[16], [17], [18], [19]]. Cadmium’s role as a metalloestrogen has been extensively studied because it accumulates in the body due to its poor excretion rate, and therefore may be harmful even at low exposures [2]. These studies have shown that cadmium promotes activation of hormone-regulated genes [20,21], proliferation of estrogen-dependent breast cancer cells [20,[22], [23], [24]], premature growth and development of mammary glands, and increased uterine weight owing to proliferation of the endometrium [2]. Although the effects of cadmium on breast cancer have been widely studied, investigations into mercury’s effects on breast cancer are limited.

Mercury exists in the environment in three forms: elemental mercury, inorganic mercury (mercuric mercury), and organic mercury (ethylmercury and methylmercury) that differ in their metabolism and toxicity [25,26]. These different forms arise from the global cycle of mercury. Elemental mercury, or mercury vapor, is a monatomic gas that evaporates from soil and water. This mercury vapor can also be emitted by volcanoes or coal-burning power stations. After about a year, the mercury vapor is converted into soluble inorganic mercury (Hg^2+^) and deposited into the earth in rain water. At this point, the inorganic mercury can be converted back into the vapor form by microorganisms or it can attach to aquatic sediments and be converted into methylmercury (MeHg) by microbes. Once in the MeHg form, it enters the aquatic food chain and becomes highly concentrated over time in large predatory fish [25].

Methylmercury (MeHg) is prevalent in the environment. The main sources of possible exposure to MeHg include occupational exposure and eating fish or wild game near the top of the food-chain that have accumulated mercury in their tissues [27]. MeHg exposure can lead to many diseases and disorders due to its liposolubility and its affinity for endogenous sulfur and selenium. When humans digest mercury-contaminated food, MeHg is absorbed in the duodenum, where it binds to thiol (R-SH) and selenol (R-SeH) groups, which are products of digestive breakdown [25].

Several groups have shown that treatment of breast cancer cells with low concentrations of mercuric chloride promotes the proliferation of these estrogen-responsive cells [5,11,14]. One other group has investigated the effects of MeHg on breast cancer cells [28]. Here, we investigated MeHg’s proliferative versus toxic effects on MCF7 breast cancer cells. We hypothesized that when breast cancer cells are cultured in the presence of MeHg concentrations comparable to physiological concentrations of estrogen, there will be an increase in cell proliferation. Conversely, we presumed that culturing cells in the presence of elevated concentrations of MeHg would promote apoptosis. These studies will bring us closer to developing therapeutic strategies for treating MeHg-induced breast cancer and will help us to take steps towards preventative interventions.

## 2 Methods

### 2.1 Cells and culture conditions

Estrogen receptor-positive MCF7 human epithelial breast cancer cells originating from an invasive ductal carcinoma of the breast, a gift from the Filardo lab at Brown University (Providence, RI), were maintained in phenol red-free Dulbecco’s Modified Eagle’s Medium (DMEM)/F-12 containing HEPES and L-glutamine with 5% fetal bovine serum. Cultures were maintained in 5% CO_2_ at 37 °C.

### 2.2 MeHg treatment and cell proliferation assay

MCF7 cells (10^4^/well) were seeded into 12-well plates in phenol red-free DMEM/F-12 HEPES with 5% FBS and incubated for 24 h. Following incubation, cells were washed 1x with Hank’s Balanced Salt Solution (HBBS) and then treated in quadruplicate with various concentrations of MeHg (0, 1 nM, 10 nM, 100 nM, 1 μM, 100 μM, 1 mM, 100 mM) delivered in HBBS for 15 min at 5% CO_2_ and 37 °C. The MeHg solution was then removed and DMEM/F-12 HEPES with 5% FBS was added back. Cells were incubated for 5 days. On day 5, cells were washed 1× with HBBS, lifted with 1x trypsin, centrifuged, and resuspended in DMEM/F-12 HEPES with 5% FBS. Cells were counted using a hemocytometer.

### 2.3 Annexin-V/PI assays

To determine apoptotic rates of MeHg-treated cells, MCF7 cells were treated with MeHg as described above. On day 5, the apoptotic rates were determined with an Annexin-V-Fluos staining kit according to the manufacturer’s instructions (Sigma-Aldrich, United States). In short, the cells were incubated in Annexin-V-FITC (Sigma-Aldrich, United States) for 30 min, washed 3x with HBBS, and analyzed by immunofluorescence [29].

### 2.4 Mercury partitioning experiment

An additional experiment was performed to examine how the treatment medium influences MeHg partitioning. Cells were treated in triplicate with 0 or 1 μM MeHg delivered in HBBS or in DMEM/F-12 HEPES with 5% FBS for 15 min at 5% CO_2_ and 37 °C. The treatment solutions (supernatants) were then removed and saved for Hg analysis. Cells were washed 1x with HBBS, lifted with 1× trypsin, and centrifuged. Cell pellets were re-suspended in 1 ml of HBBS to produce cell suspensions for Hg analysis.

Total mercury concentrations in supernatants and cell suspensions were determined using acid digestion and BrCl oxidation [30]. Briefly, 0.5-mL aliquots were digested overnight in 4 mL of 4.6 M HCl at 60 °C. After digestion, 0.4 mL of BrCl was added to the digestates; a persistent yellow color indicated complete oxidation to Hg^2+^. Just prior to analysis, excess BrCl was quenched by addition of hydroxylamine hydrochloride. Digestates were analyzed for total mercury via SnCl_2_ reduction, gold amalgamation, thermal desorption and CVAFS detection [31,32]. Controls were used as procedural blanks.

### 2.5 Statistical analysis

All of the results are presented as mean +/- standard deviation of biological triplicates or greater. Results were compared by one-way ANOVA and a probability value of p < 0.05 is considered significant.

## 3 Results

### 3.1 Low concentrations of MeHg induce MCF7 cell proliferation

**Fig. 1:**
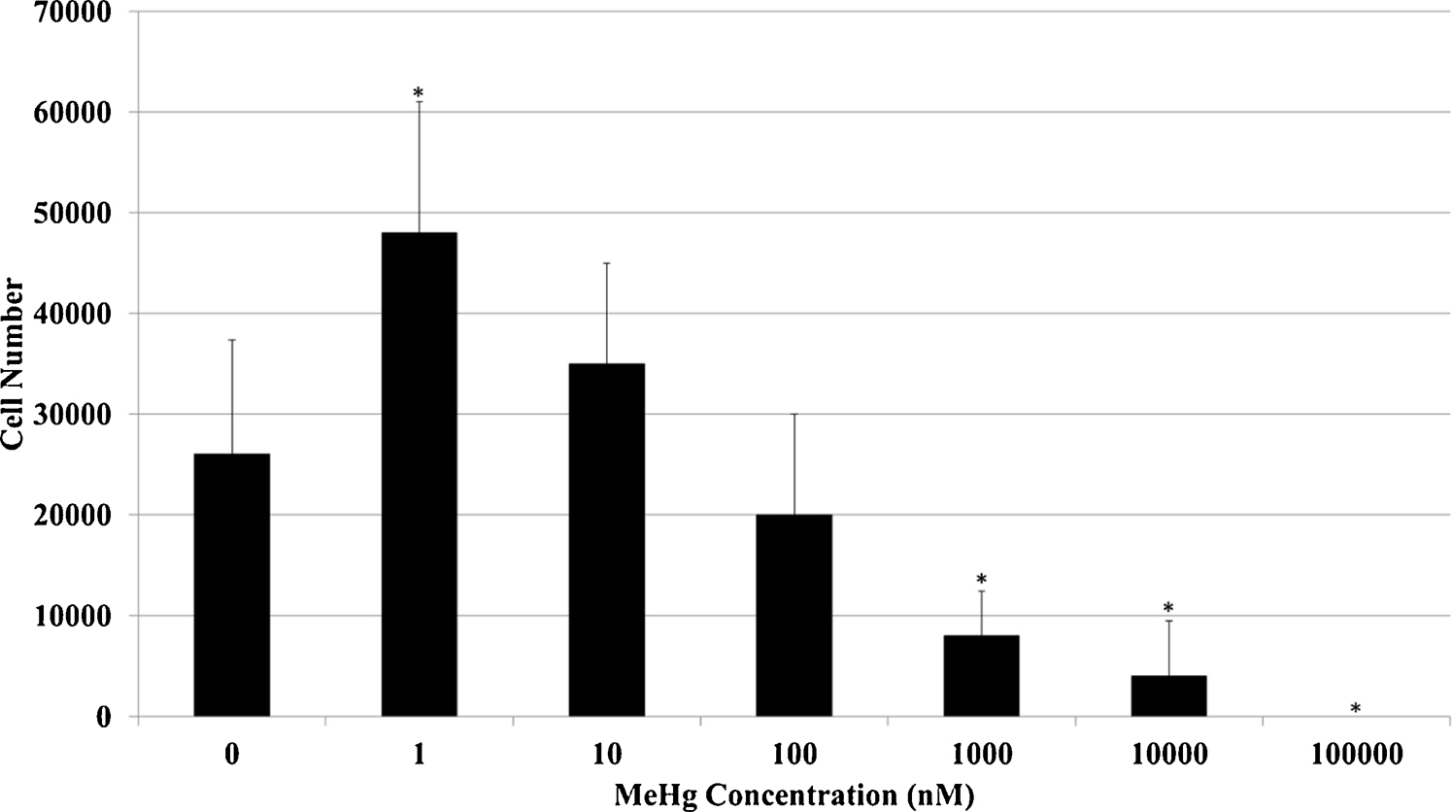
MeHg-induced proliferation and cell death in ER-positive MCF7 breast cancer cells. Asterisks indicate a significant difference (p < 0.02) in cell number relative to control (MeHg = 0 nM).

To determine the effects of MeHg treatment on cell proliferation, MCF7 human breast cancer cells were treated with concentrations of MeHg ranging from 1 nM to 100 μM. We observed significantly reduced growth at MeHg concentrations of 1 μM (p = 0.01), 10 μM (p = 0.005), and 100 μM (p = 0.001) compared to untreated cells (Fig. 1). Increased cell numbers were observed in cells treated with 1 nM MeHg (p = 0.02) when compared to untreated cells (Fig. 1). Although not significant (p = 0.26), there was a trend towards more proliferation of cells treated with 10 nM MeHg than untreated cells (Fig. 1). These results suggest that at lower concentrations, MeHg promotes proliferation, while at higher concentrations, MeHg promotes cell death.

### 3.2 High concentrations of MeHg induce MCF7 cell death

**Fig. 2:**
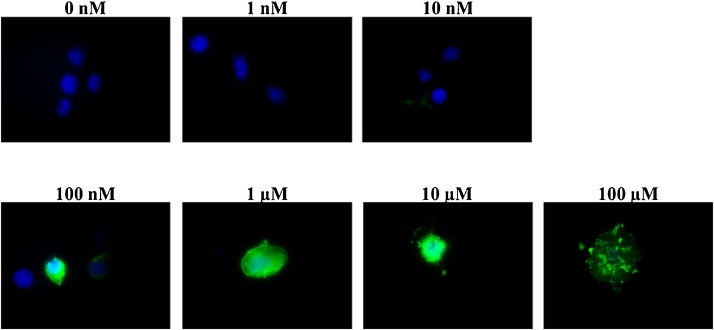
MeHg-induced apoptosis in MCFC breast cancer cells determined by Annexin-V staining 5 days following treatment. Greater staining (green) indicates apoptotic cells.

To determine whether MeHg treatment of breast cancer cells promotes apoptosis, MCF7 human breast cancer cells were treated with concentrations of MeHg ranging from 1 nM to 100 μM. Apoptosis was detected by immunofluorescence following the use of an Annexin-V-FITC staining kit. We did not observe apoptosis of cells treated with 1 nM and 10 nM MeHg, while we did observed an increase in apoptosis of cells treated with 100 nM and 1 μM. All cells treated with 10 μM and 100 μM MeHg were apoptotic (Fig. 2). These results support the proliferation assay results and suggest that at lower concentrations, MeHg does not induce apoptosis, while at higher concentrations, MeHg promotes cell death.

### 3.3 MeHg partitioning experiment

**Fig. 3:**
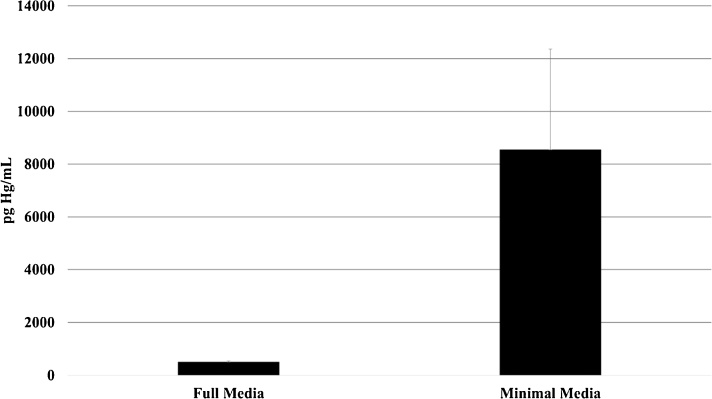
Mercury concentration in cell suspensions after treatment with 1 mM MeHg in complete versus minimal media. Cells were seeded at 10,000 cells/mL prior to treatment. Concentrations are significantly different between media at the 95% confidence level.

**Fig. 4:**
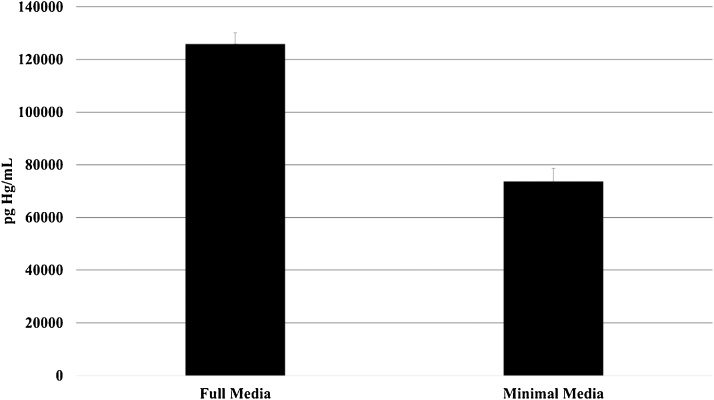
Mercury concentration in supernatants following cell treatment with 1 μM MeHg in complete media versus minimal media. Concentrations are significantly different between media at the 95% confidence level.

To compare partitioning of MeHg between cells and media, treatment with 1 μM MeHg was performed in parallel in HBBS or in DMEM/F-12 HEPES, and Hg concentrations were measured in resulting supernatants and cell suspensions. The average mercury concentration in HBBS cells suspensions was 20 times higher than the concentration in DMEM cell suspensions (Fig. 3). On the other hand, HBBS supernatant Hg concentration was about half that of DMEM supernatant (Fig. 4). These differences were significant at the 95% confidence level, indicating much greater partitioning of MeHg to cells during treatment in HBBS.

## 4 Discussion

The high incidence of hormone-related cancers may be due, in part, to the presence of environmental estrogens [5]. Cadmium, selenite, arsenite, colbolt, copper, nickel, chromium, lead, mercury, tin, and vanadate have all been shown to promote estrogen-like activity in breast cancer cells [5]. Here, we show that the treatment of ER-positive breast cancer cells with 1 nM MeHg induces cell proliferation, while we observed apoptotic cell death in cells treated with 100 nM MeHg. To our knowledge, we are the first group to demonstrate MeHg-induced proliferation of breast cancer cells at this low concentration. We investigated MeHg’s effects on breast cancer because the most common route of Hg exposure for the general population is through MeHg in fish. Furthermore, MeHg is of interest because it is known to induce oxidative stress and result in an increase in reactive oxygen species, which are often enhanced in cancer [33].

The proliferative effects of other forms of Hg have previously been investigated in MCF7 breast cancer cells (Table 1). For example, Choe et al. observed the proliferation of breast cancer cells treated with 1 μM mercuric chloride [14]. The reported relative proliferative effect of mercuric chloride (100 times the ratio between cell yield obtained with mercuric chloride and with 17β-estradiol) was 16.0%. For comparison, the relative proliferative effect of cadmium chloride was 59.7% in the same study. Martin et al. also treated cells with 1 μM mercuric chloride and reported a 2- to 5-fold increase in cell number when compared to untreated cells [5] Zhang et al. treated cells with concentrations of mercuric chloride ranging from 1 pM to 10 μM [11]. They observed significant proliferation of cells treated with concentrations of mercuric chloride ranging from 1 nM to 10 μM. They observed the highest increase in cell number, 3 times greater than in the control, in cells treated with 100 nM mercuric chloride. Egiebor et al. treated cells with concentrations of mercury (II) nitrate ranging from 0.3 μg/ml to 21.7 μg/ml [15]. They did not observe any effect on cell viability or cell proliferation, but they did observe the inhibition of cell growth in cells treated with 21.7 μg/mL mercury (II) nitrate (approximately 70 μM). The discrepancies in these results relative to our own may be due to differences in the form of mercury (inorganic vs methylmercury) used for treatment. In particular, we may have observed effects (proliferation or death) at lower concentrations because MeHg is better able to pass into cells and/or has stronger proliferative and toxic effect than mercuric ion.

**Table 1:** Summary of studies investigating the effects of mercury species on MCF7 breast cancer cells.

Study	Treatment Medium	Concentration Range and Hg species	Concentrations Causing Proliferation	Concentrations Causing Cell Death
Choe et al. [14]	DMEM with 5% bovine calf serum	1 μM mercuric chloride	1 μM	Not observed
Martin et al. [5]	IMEM with 5% fetal calf serum	1 μM mercuric chloride	1 μM	Not observed
Zhang et al [11]	RPMI-1640 medium with 10% bovine calf serum	1 pM–10 μM mercuric chloride	1 nM –10 μM	Not observed
Egiebor et al. [15]	MEM with 10% fetal bovine serum	1 μM–70 μM mercury (II) nitrate	Not observed	Not observed; growth inhibited at 70 μM
Sukocheva et al. [28]	DMEM with 5% bovine calf serum	10 nM–100 μM methylmercury	0.5 μM–1 μM	5 μM–100 μM
This study	Hank’s Balanced Salt Solution	1 nM–100 μM methylmercury chloride	1 nM	100 nM– 100 μM

Abbreviations- DMEM (Dulbecco’s Modified Eagle’s Medium, IMEM (Improved Minimal Essential Medium), RPMI-1640 (Roswell Park Memorial Institute), MEM (Modified Essential Medium).

One group observed the stimulation of growth of MCF7 cells in the presence of 0.5–1 μM MeHg, which is 1000-fold greater than the MeHg concentration that stimulated the proliferation of the same cell line in our experiments [28]. Different treatment conditions used in the studies on mercury’s effects on breast cancer cell could also be responsible for the variations in results. Choe et al. [14], Egibor et al. [15], Martin et al. [5], Zhang et al. [11], Sukocheva et al. [28], and our group all used MCF7 breast cancer cells, but employed different treatment protocols (Table 1). Treatment conditions are important because metals are known to interact with amino acids to promote local folding [[34], [35], [36], [37]], and proteins are present in many cell culture media formulations. Specifically, inorganic mercury and MeHg bind to thiol-containing proteins, such as glutamine, cysteine, and albumin [26], which are proteins that are present in some cell culture media formulations. The binding of mercury to these proteins in the media may prevent the association of mercury with cells and account for the fact that other groups observed proliferative effects at higher concentrations of Hg than we did. In fact, we observed cell death at concentrations where others observed proliferation. Each of the other study protocols involved treating cells in medium supplemented with serum, whereas we treated cells in the presence of HBSS in the absence of any proteins (Table 1).

Our partitioning experiment shows that much more MeHg is associated with cells that are exposed in HBBS medium compared to DMEM medium with FBS. The large difference in partitioning suggests greater availability of MeHg when treatment occurs in medium that is free of proteins and amino acids. MeHg is known to bind strongly to organic matter that contains reduced sulfur groups; for example, stability constants for MeHg with organic matter via the reaction: RS-+ CH_3_Hg^+^→CH_3_HgSR have been measured in the range of 10^15^–10^20^ [38,39]. Previous studies have revealed that MeHg can be taken up as MeHg-cysteine via the neutral amino acid transporter [[40], [41], [42]], although this is not the sole transport mechanism that has been observed in all cell lines. For example, MeHg-Cl complexes can also enter cells via passive diffusion [41,43]. The fact that greater uptake occurred in our experiment in the absence of amino acids suggests that MeHg entered via diffusion of neutral inorganic species. In the complete medium, complexation with large organic molecules that don’t effectively cross the lipid membrane could have lowered uptake of MeHg. Alternatively, competitive inhibition by methionine or other organic compounds could have decreased uptake by the neutral amino acid transporter [42,43]. Regardless of the uptake mechanism, our results support the idea that cell proliferation or death occurred at lower than previously observed levels because we treated our cells in minimal medium.

The proposed mechanism for MeHg-induced cell proliferation, in the absence of thiol-containing proteins from full cell culture media, involves MeHg entry into the cell via passive diffusion of neutral MeHg-inorganic complexes (MeHg-Cl). Once inside the cell, it is proposed that MeHg binds to the hormone-binding domain of the ER. It is also possible that MeHg is demethylated to form inorganic mercury before binding to the ER, as it has previously been shown that inorganic mercuric chloride binds with high affinity to the hormone-binding domain of the ER [5]. It is important to note that our method of mercury analysis did not distinguish between mercury forms. Following mercury binding to the ER, the activated ER localizes in the nucleus, dimerizes, and binds to an estrogen response element to promote cell proliferation [4].

## 5 Conclusion

To our knowledge, this is the first study to show that 1 nM MeHg promotes the proliferation of breast cancer cells. We also showed that more MeHg associates with cells that are exposed in minimal media compared to full media with FBS, offering a possible explanation for the fact that we observed proliferation at lower concentrations of mercury than other groups and that we observed cell death at mercury concentrations where others observed proliferation. Further studies may investigate the proliferative effects of MeHg on other cell types and in comparison to estrogen. Future studies may also investigate the possible demethylation of MeHg before binding to the ER. Finally, future studies may also investigate the proliferative effects of MeHg in an *in vivo* model.

## Declaration of interest

The authors report no declarations of interest.
